# Sequence dependency of canonical base pair opening in the DNA double helix

**DOI:** 10.1371/journal.pcbi.1005463

**Published:** 2017-04-03

**Authors:** Viveca Lindahl, Alessandra Villa, Berk Hess

**Affiliations:** 1 Department of Physics and Swedish e-Science Research Center, KTH Royal Institute of Technology, Stockholm, Sweden; 2 Science for Life Laboratory, Stockholm and Uppsala, Stockholm, Sweden; 3 Department of Biosciences and Nutrition, Karolinska Institutet, Huddinge, Sweden; Baltimore, UNITED STATES

## Abstract

The flipping-out of a DNA base from the double helical structure is a key step of many cellular processes, such as DNA replication, modification and repair. Base pair opening is the first step of base flipping and the exact mechanism is still not well understood. We investigate sequence effects on base pair opening using extensive classical molecular dynamics simulations targeting the opening of 11 different canonical base pairs in two DNA sequences. Two popular biomolecular force fields are applied. To enhance sampling and calculate free energies, we bias the simulation along a simple distance coordinate using a newly developed adaptive sampling algorithm. The simulation is guided back and forth along the coordinate, allowing for multiple opening pathways. We compare the calculated free energies with those from an NMR study and check assumptions of the model used for interpreting the NMR data. Our results further show that the neighboring sequence is an important factor for the opening free energy, but also indicates that other sequence effects may play a role. All base pairs are observed to have a propensity for opening toward the major groove. The preferred opening base is cytosine for GC base pairs, while for AT there is sequence dependent competition between the two bases. For AT opening, we identify two non-canonical base pair interactions contributing to a local minimum in the free energy profile. For both AT and CG we observe long-lived interactions with water and with sodium ions at specific sites on the open base pair.

## Author summary

The DNA double helix, a molecule that stores biological information, has become an iconic image of biomedical research. In order to use or repair the information it carries, the bases that are stacked in the helix need to be chemically exposed. This can happen either by separating the two strands in the helix or by flipping out individual bases. Here, we focus on the latter process. Usually proteins are involved in interactions with bases, but it is still unclear if bases are pulled out actively by proteins or if they act on spontaneously flipped bases. Although experiments can detect base pair opening, it is difficult to detect which base moves in which direction. Here, we present results from molecular dynamics simulations using a recently developed sampling method which improves the statistics in the simulations by enhancing the probability of the base pair opening event. We observe differences in probability, modes and mechanism of opening that depend not only on the types of the bases in the pair, but also strongly on their neighbors. This provides essential information for understanding how DNA functions.

## Introduction

DNA base pair opening, or base breathing, is the process of breaking the hydrogen bonds of a base pair. Opening is the first critical step of base flipping in which either base moves away from the DNA double helix. Fundamental biological processes such as DNA replication, modification and repair [1–3] rely on this mechanism for accessing the functional groups of the bases.

Experimentally, X-ray crystallography has revealed how enzymes directly operate on a flipped-out DNA base by binding it into the enzyme active site [1]. Furthermore, nuclear magnetic resonance (NMR) experiments have quantified base pair opening in terms of base pair lifetimes and free energies by measuring imino proton, i.e. H1 in guanine and H3 in thymine, exchange rates with solvent [4, 5]. The free energy of the open, proton exchanging state relative to the closed state is calculated by relying on a two-state model. Most importantly, these NMR studies have proven that base pair opening occurs spontaneously and without help from an enzyme on a timescale of milliseconds [6]. Recently, the dynamics of base flipping has also been studied using fluorescence correlation spectroscopy (FCS) [7].

The exact mechanism for base flipping is still not well understood on an atomistic level [8]. In the presence of an enzyme, protein-DNA interactions may be an important first step in the process [9]. Alternatively, spontaneous base pair opening could be the trigger of further enzyme interactions [10]. In either case, characterizing base pair opening in terms of DNA only is necessary for fully understanding the more complex scenario of DNA in an enzyme environment. Specifically, the question of how DNA base pair sequence and helical conformation affect the propensity for base pair opening remains largely unanswered.

Early studies [11] agree that the characteristics of opening is primarily determined by the base pair type. The lifetimes of the canonical Watson-Crick (WC) base pairs AT and GC have been observed to be 1–5 and 10–50 ms, respectively. However, the nucleic acid sequence and helical conformation are also important factors [12, 13]. For example, AT base pairs in so called A-tracts have closed state lifetimes of up to 100 ms [13, 14].

Because of the short lifetimes of the open states, experimental studies have limited resolution. The problem of mapping laboratory measurements to the underlying molecular event is highlighted by a recent FCS study [7] on DNA mismatches in which the observed base pair lifetimes were significantly longer than those measured by NMR. The straightforward conclusion is that NMR and FCS measurements are not directly comparable; while FCS is sensitive only to extrahelical flipping, NMR can detect any opening angle large enough for solvent to gain access to the bases.

To gain further atom-level insight into the flipping process, a substantial amount of computational effort has been put into characterizing the opening pathway in recent decades [8]. Because of the high free energy barriers involved in the base pair opening, biased simulation algorithms are required for efficient sampling. The method of choice has typically been molecular dynamics (MD) combined with umbrella sampling along a reaction coordinate, i.e. a function of the coordinates. More recently, adaptive biasing methods have been combined with principal component analysis [15] and path optimization methods [16].

In many cases the results of these studies are in qualitative but not quantitative agreement with each other [8]. In addition, making direct comparisons or drawing an overall conclusion about the source of a discrepancy is in practice very difficult because typically different authors have studied different DNA sequences and base pairs using different or modified force fields, reaction coordinates and sampling methods. Furthermore, since the first papers on this topic were published, new simulation methods have appeared, force fields have been developed further and computational power has continued to grow. Indeed, recent very long MD simulations indicate that the structure and dynamics of the DNA duplex requires *μ*s of simulation time to fully converge [17]. In summary, there is still a great need for more systematic and extensive computational studies.

Here, we calculate free energies and structurally characterize the opening of canonical DNA base pairs. We use a newly developed sampling algorithm, the accelerated weight histogram method (AWH) [18]. In contrast to umbrella sampling, AWH samples multiple transition pathways within one simulation which is a good test for hysteresis effects. To probe sequence dependency, we present results for 11 target base pairs of type GC and AT located in different positions in either of two DNA sequences. As a test of existing force fields and of the simulation method we use two different force fields, CHARMM27 [19] and parmbsc1 [20], and compare calculated free energies to experimental values obtained from NMR.

## Methods

### Choice of reaction coordinate

As a first step in studying base pair opening we need a reaction coordinate that parameterizes the opening pathway. Our use of the reaction coordinate is two-fold. First, we apply a bias along this degree of freedom to promote opening and escape the WC state, i.e. conformations where the WC hydrogen bonds are intact. Second, we use it to analyze and understand the resulting data.

**Fig 1:**
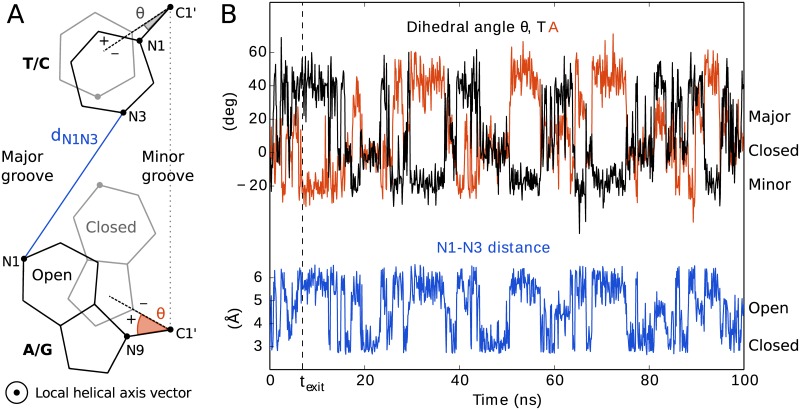
Enhanced sampling of base pair opening by adaptively biasing along *d*_N1N3_. The opening of a base pair can be described either by the distance coordinate *d*_N1N3_ or by two dihedral angles *θ*, one for each base (A). The closed state (gray configuration) is characterized by *θ* ≈ 0 and small *d*_N1N3_. Open configurations (black) have larger *d*_N1N3_ values and negative or positive *θ* values, depending on the direction of opening. Here, we improve sampling of the opening event by applying a bias along *d*_N1N3_ using the adaptive biasing method AWH. Example trajectories are shown for one such simulation, for the case of opening of an AT base pair (B). Both *θ* angles are sampled simultaneously, according to their Boltzmann distribution at each given value of *d*_N1N3_. After exiting an initial stage of the method (dashed vertical line), sampling of *d*_N1N3_ is expected to be roughly uniform and its dynamics diffusive.

In most previous work, authors have used geometrically intuitive measures. Early work [21] used the distance between the N1 atom of the purine (base A or G) to the N3 atom of the pyrimidine (base T or C) defining the central WC hydrogen bond of the opening base pair, see Fig 1A. We denote it *d*_N1N3_. More recently, different variants of dihedral angles describing the position of the opening base with respect to the helical axis have been the most popular choice [22–24] mainly because they distinguish between which base flips into which groove of the DNA helix, major or minor. We have chosen one such dihedral angle [22] for analysis purposes in this work. We further shift this angle by the average angle in the WC state such the closed state has angles of approximately zero, the major groove has positive angles and the minor groove negative angles. We denote the resulting dihedral angle *θ*, see Fig 1A.

In this study we prefer to bias along *d*_N1N3_ rather than a dihedral angle. Our primary aims are to investigate sequence dependency of the opening free energy and to be able to compare our results to NMR data. NMR experiments measure the rate of imino proton exchange with solvent, a rare event which can occur as soon as the central WC hydrogen bond is broken and the imino proton is exposed to the surrounding solvent. A straightforward way of sampling such configurations in simulations is to bias sampling toward larger values of *d*_N1N3_. Values of *d*_N1N3_ ≾ 3 Å characterize the WC state, whereas the value of a dihedral angle is not as specific in mapping to this state [23]. Indeed, high “sensitivity” to *d*_N1N3_ has even been used as a measure of a “good” dihedral reaction coordinate [24]. In addition, pathway optimization studies [16] indicate that *d*_N1N3_ “elongation” is an important, previously overlooked, component in the early stages of opening.

Here, we wish to sample the most probable pathways of the opening process and would rather not add the complication of specifying which base should go in which direction. In addition, forcing each base separately into both the major and the minor groove is likely computationally less efficient when there is a preference for one base and/or direction. Thus, choosing *d*_N1N3_ as a biasing coordinate allows us to connect simulations to experiments while making minimal assumptions on the preferred pathway.

In previous work [15, 23, 25], the focus has typically been on characterizing the full extent of base flipping. Although the base is very likely to proton exchange for large flipping angles, the free energies of such states are expected to be higher than proton exchanging states with smaller angles and are thus not expected to contribute significantly to NMR measurements. For efficiency reasons we therefore sample distances large enough to expose the imino proton but small enough to avoid irrelevant, high free energy states.

### AWH adaptive biasing

In the popular umbrella sampling method [26] sampling along the reaction coordinate pathway is ensured by running multiple simulations and harmonically restraining each simulation to sample around a particular reaction coordinate value, *λ*. The unbiased free energy profile is subsequently calculated by reweighting and combining samples from all simulations using the weighted histogram analysis method (WHAM) [27]. The main drawback of umbrella sampling is that the umbrella restraints may introduce ergodicity problems. Specifically, the results can be very sensitive to the starting configurations since sampling is essentially restrained to a single pathway. Thus, if applied naively, the free energy profiles from umbrella sampling may seem converged while in fact important pathways have effectively been excluded.

AWH [18, 28] is similar to umbrella sampling in that it applies harmonic restraints along a reaction coordinate. There are fundamental differences however. First of all, instead of each simulation being assigned a single *λ* value, one AWH simulation samples all *λ* values. Fig 1B shows an example trajectory where *λ* corresponds to *d*_N1N3_. This mitigates ergodicity problems by allowing one trajectory to explore multiple pathways. Again this is examplified by the figure, which shows one trajectory that samples both pathways where A opens toward the major groove and T toward minor, as well as the the other way around. Second, with umbrella sampling the bias of each simulation is constant and the overall bias is implicitly set by the mapping of simulations to *λ* values. With AWH on the other hand, the bias is explicitly included in each simulation as a time-dependent function of *λ*.

More specifically, AWH samples an extended, time-dependent ensemble *P*(*x*, *λ*; *t*), where the probability of each *λ* is set and tuned by a bias function *g*(*λ*; *t*), *P*(*x*, *λ*; *t*)∼*e*^*g*(*λ*;*t*)^. Configurations *x* are sampled using MD and *λ* is sampled using a Gibbs sampler, i.e. *λ* is regularly drawn from *P*(*λ*|*x*). The bias function is updated at regular intervals by increasing the bias in undersampled regions according to the following formula

$$\begin{array}{c} \Delta g\left(\lambda ;t\right)=-\ln\frac{W_{\text{ref}}\left(\lambda ;t\right)+W_{\text{sampled}}\left(\lambda ;t\right)}{W_{\text{ref}}\left(\lambda ;t\right)+W_{\text{target}}\left(\lambda ;t\right)}, \end{array}$$

where *W*_sampled_(*λ*;*t*) = ∑_*t*′′<*t*′<*t*_ *P*(*λ*|*x*(*t*′)) is the sum of probability weights sampled since the last update at time *t*′′ *W*_target_(*λ*; *t*) is a user-defined target distribution function with ∑_*λ*_ *W*_target_(*λ*; *t*) = ∑_*λ*_ *W*_sampled_(*λ*; *t*) (here chosen uniform); and *W*_ref_(*λ*; *t*) is a reference weight histogram representing all prior sampling history. As samples accumulate over time, *W*_ref_(*λ*; *t*) grows and in the long time limit, Δ*g*(*λ*; *t*)∼1/*W*_ref_(*λ*; *t*)∼1/*t*. Thus, the fluctuations in the bias closely connects to the current amount of sampling.

The time-dependent bias alters the dynamics of the simulations. Initially, when *W*_ref_ is relatively small, large bias updates push the system out of local free energy minima, promoting exploration along the reaction coordinate. Later, when *W*_ref_ has grown, the bias changes slower and the dynamics becomes increasingly diffusive as the bias converges. Clearly then, the growth rate of *W*_ref_ is key for the efficiency and convergence of the method. Initially, the bias is still far from optimal and sampling is statistically inefficient, i.e. correlation between samples is high. Thus, for sake of robustness and efficiency of the method it is necessary to restrict the histogram growth initially. Here we follow the scheme motivated and demonstrated in previous work [18]. Briefly, the AWH simulation is divided into two stages: an initial stage where *W*_ref_ grows exponentially but slower than the real sampling rate, and a final stage, *t* > *t*_exit_, where *W*_ref_ grows linearly according to the sampling rate. The transition from initial to final stage is defined such that in the final stage the size of *W*_ref_ equals the actual number of collected samples.

The slower growth of *W*_ref_ in the initial stage corresponds to continuously scaling down the histogram, effectively assigning more weight to later samples than earlier ones. In the final stage each sample is given equal weight. When analyzing data from AWH simulation we propose to weigh samples in the same way. Here, we simply ignore initial stage data and perform all free energy calculations on final stage data only, for the sake of simplicity and because initial stage samples may anyway be far from equilibrium. This is not critical since in this work most of the data in each run is in any case sampled from the final stage, see Fig 1B.

### Calculating free energies from biased trajectories

From data biased along a reaction coordinate *ξ* we would like to extract an estimate of the unbiased free energy *Φ*(*u*) for any observable *u* (including the case *u* = *ξ*). This is easily done post-simulation by reweighting the biased samples as (see S1 Appendix for details)

$$\begin{array}{cc} e^{-\hat{\Phi }\left(u\right)} & =\sum_{i,t}1_{u}\left(u_{t}^{i}\right)e^{-b_{t}^{i}\left(\xi_{t}^{i}\right)}\int \;\;d\xi^{\prime }\;e^{-\hat{\Phi }\left(\xi^{\prime }\right)+b_{t}^{i}\left(\xi^{\prime }\right)}, \end{array}$$ (1)

where $\hat{\Phi }\left(u\right)$, $\hat{\Phi }\left(\xi \right)$ are free energy estimates along *u* and *ξ*, respectively. The index *i* runs over independent AWH simulations with observables $u_{t}^{i}$ at time *t*, each with its own effective bias $b_{t}^{i}\left(\xi \right)$ applied. 1_*u*_ is shorthand for the required binning procedure: $1_{u}\left(u_{t}^{i}\right)=1$ if $u_{t}^{i}$ falls into the bin labeled by *u* and 0 otherwise. The unbiasing is taken care of by the factor $e^{-b_{t}^{i}\left(\xi_{t}^{i}\right)}$. This factor is further properly normalized by the integral over *ξ* (the partition function of the extended ensemble). This expression is exact for equilibrium sampling which is a reasonable approximation in the AWH final stage. In this work, in order to simplify the analysis slightly, we have further applied the approximation that the bias is constant in the final stage, i.e. $b_{t}^{i}\approx b_{t_{\text{final}}}^{i}$.

When *u* = *ξ*, Eq (1) needs to be solved self-consistently since now the sought-for variable occurs on both sides of the equation. However, our AWH implementation already calculates and outputs an estimate ${\hat{\Phi }}^{i}\left(\xi \right)$ for each simulation *i* on the fly [18]. This is convenient because no post-processing is needed and does not require frequently writing to disk. Data from multiple simulations is therefore combined by self-consistently solving the following equation for the combined estimate $\hat{\Phi }\left(\xi \right)$,

$$\begin{array}{cc} e^{-\hat{\Phi }\left(\xi \right)} & =\sum_{i}N^{i}e^{-{\hat{\Phi }}^{i}\left(\xi \right)}\frac{\int \;\;d\xi^{\prime }\;e^{-\hat{\Phi }\left(\xi^{\prime }\right)+b^{i}\left(\xi^{\prime }\right)}}{\int \;\;d\xi^{\prime }\;e^{-{\hat{\Phi }}^{i}\left(\xi^{\prime }\right)+b^{i}\left(\xi^{\prime }\right)}}, \end{array}$$ (2)

where *N*^*i*^ are the number of samples collected in simulation *i* and *b*^*i*^(*ξ*) has been evaluated at *t* = *t*_final_.

We estimate the standard deviation of our free energy averages, obtained either by Eqs (1) or (2), using jackknifing (details in S1 Appendix).

### Estimating the opening free energy

From the biased simulations we obtain the free energy profile along the reaction coordinate. In contrast, the free energy obtained from NMR data is that of an “open” state relative to a “closed” state. The open state is comprised of configurations that expose the imino proton to solvent, thus contributing to the measured proton exchange rate. In order to calculate a comparable free energy from simulations we therefore need an observable of solvent accessibility. The free energy corresponding to this observable is then calculated using the reweighting procedure of Eq (1).

In the past there has only been a limited number of direct comparisons of free energies from experiment and simulations [23, 29]. Typically, the approach has been to use the solvent accessible surface area (SASA) of the imino proton as the observable and labeling configurations with SASA larger than a chosen cutoff area as “open” and the rest as “closed”. A drawback of using SASA is that it strongly depends on the chosen cutoff area and is fairly slow to calculate for large data sets.

In our simulations we consider the imino proton to be solvent accessible if it is hydrogen bonded to a water molecule. The hydrogen bond is defined using an acceptor-donor distance cutoff of 3.5 Å and an acceptor-donor-hydrogen angle cutoff of 30°. Here, we refer to configurations with such a hydrogen bond as “open” and the corresponding free energy as the “opening free energy”, or the “calculated opening free energy” to distinguish it from the opening free energy obtained experimentally. We have verified that this opening free energy is very similar to the free energy obtained using SASA with a small cutoff area (∼0.1 Å^2^).

It is important to note that our solvent accessibility criterion is not expected to exactly capture the open state detected by NMR. Indeed, an exact mapping of a given configuration to “open” or “closed” as inferred from NMR data does not exist. The NMR open state is only implicitly defined by a two-state model parameterized by experimentally determined average rates. Notably, it is assumed that the open state has approximately the same proton transfer rate as a free nucleotide [6] while realistically there could e.g. be partially open states which behave differently. Furthermore, the experimental free nucleotide reference state includes configurations that are not hydrogen bonded which our accessibility criterion will exclude. This overestimates the free energy by up to −ln 0.8 = 0.2 *k*_*B*_ *T* relative to the NMR free energy since we observe hydrogen bonding 80-90% of the time in the free nucleotide and maximally open configurations. An additional complication is that the approximations made in the NMR modelling could be more or less valid for different sequences and base pairs.

### Simulation setup

**Fig 2:**
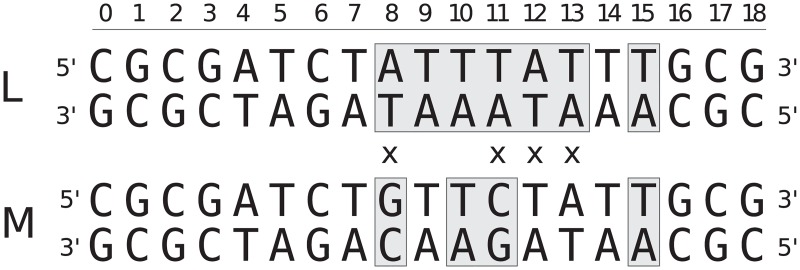
The simulated base sequences, L and M. Sequence differences are marked by ‘ × ’. Gray background indicate the target base pairs simulated in this study. The same sequences apart from the added end base pairs have been characterized in NMR experiments [6].

We have simulated two sequences, denoted L and M, that have previously been experimentally characterized using NMR experiments [6]. For each sequence we investigated the opening of several target base pairs located in the region where the sequences differ, see Fig 2. We added an extra GC base pair at the ends of each system which was restrained as described below to avoid ends effects.

Simulations were performed using a version of the GROMACS [30] master branch code [31], extended by an implementation of AWH [32]. We used the force fields CHARMM27 [19], CHARMM36 [33] and parmbsc1 [20]. CHARMM27 is available in the GROMACS package [34]. GROMACS compatible force field files for CHARMM36 were downloaded from the authors’ website [35]. Parmbsc1 for GROMACS was implemented by us [36]. Together with the CHARMM force fields we used CHARMM-modified TIP3P water [37] and sodium ions. With parmbsc1 we used SPC/E water [38] and sodium ions [39]. We list the main MD settings here but refer to the template GROMACS parameter input file in S1 File for details. Note that in this section we use GROMACS compatible units, e.g. 1 nm = 10 Å. The MD time step was 0.002 ps. Bonds involving hydrogens were constrained using LINCS [40]. The temperature was kept at 300 K using the v-rescale thermostat [41] and the pressure at 1 bar using Parrinello-Rahman pressure coupling [42, 43]. Long-range electrostatics were calculated using particle mesh Ewald [44]. For CHARMM, Lennard-Jones interactions beyond the cutoff were calculated by switching the force to zero. For parmbsc1 the force was shifted to zero and dispersion-correction was applied for energy and pressure.

The DNA starting structures were generated in the double strand B-form using the 3DNA software [45]. Each system was solvated in explicit water and neutralized by adding sodium ions. The rhombic dodecahedron simulation box had dimensions of approximately 8.7 nm, fitting ∼14800 water molecules. Base pairs at both ends of the DNA helix were restrained by 0.5 ⋅ *k*(*d*_N1N3_ − 0.3 nm)^2^, where *k* = 1000 kJ/(mol ⋅ nm^2^).

The solvated system was equilibrated by first energy minimizing (until the maximum force < 1000 kJ/mol), then adding 50 ps of NVT MD, and finally NPT equilibrating for 50 ns before the production AWH runs.

The AWH reaction coordinate sampling interval was defined by [*λ*_min_, *λ*_max_] = [0.25, 0.65] nm. The force constant at each *λ* was 32 000 kJ/(mol ⋅ nm^2^). The AWH code automatically determines the *λ* spacing base on the force constant; here Δ*λ* = 3 ⋅ 10^ − 3^ nm. As described in previous work [18], the initial bias update size is set as a function of two input parameters: an estimate of the diffusion along the biased coordinate and an estimated initial error. Here we estimated the diffusion to 5 ⋅ 10^ − 5^ nm^2^/ps and the initial error to 5 *k*_*B*_ *T*. These estimates can be very rough since the efficiency of AWH is quite robust to their values [18].

In our initial biased CHARMM27 simulations we encountered sampling problems due to configurations in which the two partner bases on opposite strands stack on top of each other. For sampling efficiency reasons we therefore added to all our simulations a bias potential designed to avoid sampling such configurations. Explicitly, we added the following potential acting on the distance between the center of mass of the six-member ring of each base: *V*_ring_ = 0.5 ⋅ *k*(*d*_ring_ − 0.48 nm)^2^ for *d*_ring_ < 0.48 nm and *V*_ring_ = 0, otherwise, where *k* = 32 000 kJ/(mol ⋅ nm^2^). The ring distance cutoff was set by first calculating the free energy landscape for the two-dimensional coordinate (*d*_N1N3_, *d*_ring_) and then determining the lowest value of the cutoff that would still exclude base pair stacked regions of phase space. See section Base pair stacked state for further details and discussion.

The AWH simulations were 100 ns and 200 ns long for CHARMM and Parmbsc1, respectively. The simulation lengths were chosen differently because we observed slower transitions along the biased coordinate for Parmbsc1 compared to CHARMM27. Each target system, i.e. combination of force field, DNA sequence and base pair to open, was replicated 16 times using different AWH and thermostat seeds, starting from the same equilibrated structure. Free energy averages and error estimates were obtained by combining these independent simulations (see S1 Appendix). Two sets of simulations were extended further in order to reach comparable free energy accuracies for all target systems: Parmbsc1 L:TA10 (576 ns) and CHARMM27 M:CG11 (222 ns).

The reweighting procedures of Eqs (1) and (2) can be sensitive to the amount of sampling due to the exponential of the reweighting factor. Therefore one can obtain higher accuracy of the free energies by excluding individual runs that have few transitions across the sampling region. Here we require at least one back-and-forth transition from *d*_N1N3_ ≤ 0.27 nm to ≥ 0.5 nm, or vice versa. This criterion excluded in total three runs one of each CHARMM27 L:TA10 (+0.03 *k*_*B*_ *T*) and M:TA15 (+0.5 *k*_*B*_ *T*); and one Parmbsc1 L:TA9 (+0.07 *k*_*B*_ *T*), where the values in parenthesis show how excluding the run affected the calculated opening free energy. In both excluded CHARMM27 runs we observed base pair stacking tendencies of the target base pair and/or of neighboring base pairs.

## Results and discussion

### Free energies

**Fig 3:**
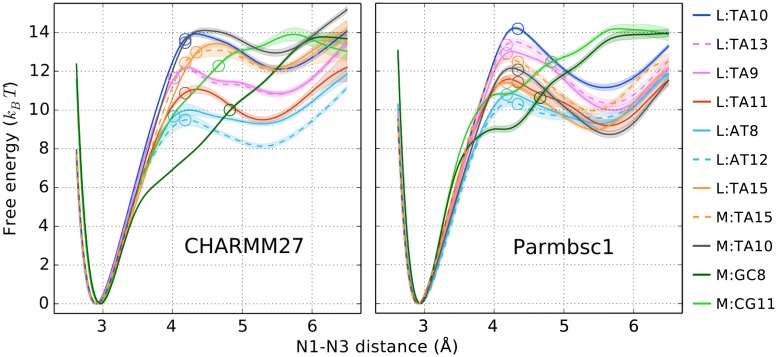
Free energy profiles along the reaction coordinate *d*_N1N3_. Continuous error bars indicate ±1*σ* (see S1 Appendix). Base pairs with the same nearest neighbors have the same color but either solid or dashed lines. Circles mark the distance where the imino proton is hydrogen bonded with water 20% of the time.

**Fig 4:**
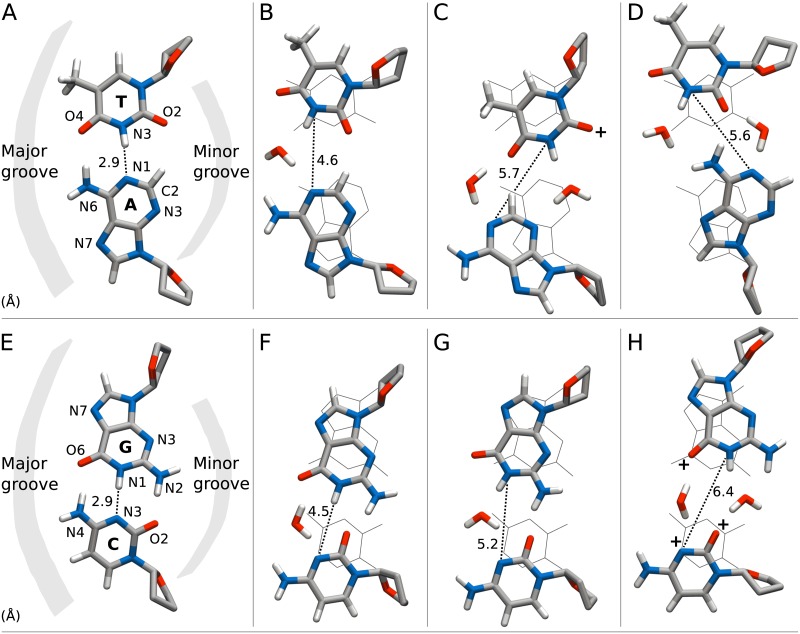
Sampled base pair configurations. Representative configurations at different *d*_N1N3_ values were chosen from Parmbsc1 L:AT12 (A–D) and M:CG11 trajectories (E–H). The distance values are in the figure, indicated by a dotted line. Carbon atoms are shown in gray, hydrogens in white, oxygens in red and nitrogens in blue. Waters within hydrogen bond distance of donor/acceptor atoms on both bases are also included to show examples of water bridges. Acceptor atoms that are very often in contact with Na^+^ in similar configurations are marked with a ’+’. Base-paired WC configurations (A), (E) are shown in line representation behind the open configurations, (B–D) and (F–H), to help visualize the extent of opening. Note that no timeline is implied from left to right.

Fig 3 shows free energies as a function of the reaction coordinate *d*_N1N3_ for CHARMM27 and Parmbsc1. Distances where the solvent accessibility reaches 20% are marked with a circle on each curve (roughly at 4.2 Å for AT and 4.8 Å for GC). This indicates the extent of opening that NMR experiments are sensitive to and which configurations we label as “open” or “closed” when calculating the opening free energies. In Fig 4, we show representative configurations for different values of *d*_N1N3_ to aid the reader in visualizing the opening structures in different regions along the reaction coordinate.

For AT pair opening there are two distinct free energy minima: the global minimum at 2.9 Å (WC state) and a local minimum at *d*_min_ ∼ 5.5 Å which is up to 2 *k*_*B*_ *T* deep for CHARMM27 and 3 *k*_*B*_ *T* for Parmbsc1. In order to characterize the local minimum we analyzed the interactions between A and T of the target base pair by counting minimum distance atom pairs for frames with *d*_N1N3_ ∈ *d*_min_ ± 0.3 Å. We found two clearly dominating base pair interactions which account for 80–90% of the configurations. First, for A opening into the major groove and T shifted toward minor the most frequent interaction was that of the A:C2 hydrogen and the major groove carbonyl oxygen O4 of T, see Fig 4C. Alternatively, for T opening toward major and A perturbed into minor, the most frequent base pair interaction was a non-WC hydrogen bond between the hydrogen of A:N6 and the minor groove carbonyl oxygen O2 of T, see Fig 4D.

Structures with the latter interaction have been suggested based on molecular mechanics simulations to be responsible for proton exchange from AT pairs [46]. The same interaction has been observed also in an earlier MD study [47], but without directly connecting it to a local minimum in the free energy profile as we do here. Interestingly, we have not found previous mention of the first interaction. In our simulations however it is clearly a relevant interaction, occurring 60–80% of the local minimum time for CHARMM27 and 50–60% of the time for certain Parmbsc1 target base pairs.

For GC pair opening the free energy increases monotonously up until *d*_N1N3_ ∼ 5.5 Å where it levels off. To investigate what happens beyond the sampled interval, in particular for GC opening, we ran simulations of Parmbsc1 L:TA11 and M:CG11 but extending the interval from 6.5 Å to 8.0 Å (and extending the simulation time to obtain comparable statistical accuracies). In both cases the average free energy profile rises 2–3 *k*_*B*_ *T* in the added interval indicating that we are not missing important states from the extended region. We note that excluding the weight of these states will lead to a systematic (positive) error in our calculated opening free energies (presented below). From these simulations we estimate this error to be ∼0.3 *k*_*B*_ *T*.

To detect the presence of next-to-nearest-neighbor sequence effects we included several target base pairs with identical nearest neighbors but different neighbors beyond that. Such related profiles have the same color but are either solid or dashed in Fig 3. In the figure it is clear that targets with the same nearest neighbors in general are more similar than those that have different direct neighbors which shows that the nearest-neighbor sequence is a dominating factor for the free energy. However, for Parmbsc1 L:TA15 vs M:TA15 the free energy profiles are clearly not overlapping and for CHARMM27 L:AT8 vs L:AT12 the profiles have similar barrier but different depths of the local minimum. This indicates the existence of other effects competing with nearest-neighbor effects.

**Fig 5:**
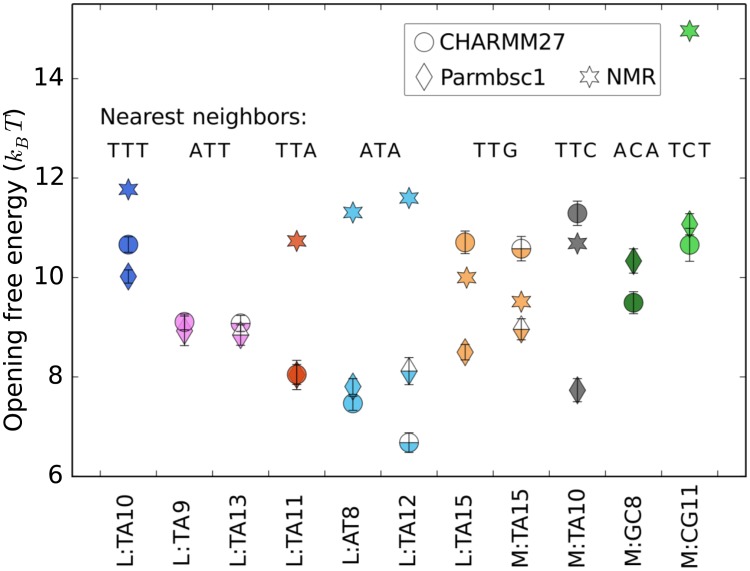
Calculated and experimental opening free energies. Base pairs are grouped according to the nearest neighbor triplet sequence, see main text for details. Note that there is no experimental data available for certain base pairs.

In Fig 5 we show the calculated opening free energies for each base pair. The color coding is analogous to Fig 3 (with half-filled markers corresponding to dashed lines). NMR free energies at the simulated temperature were obtained by interpolating between experimental values [6] at the two nearest temperatures. Error bars in all cases are 0.1–0.3 *k*_*B*_ *T*. In the figure, we have grouped base pairs based on the nearest neighbors sequence, i.e. a triplet, in direction 5′ to 3′ on the strand containing the target pyrimidine. In terms of these triplets, both force fields predict a free energy trend TTT > ATT > TTA ≥ ATA. For GC base pairs the trend is TCT > ACA. The results are not as clear when T is flanked by either G or C, i.e. for L:TA15, M:TA15 and M:TA10. In these cases, CHARMM27 in general gives roughly 2–3 *k*_*B*_ *T* higher values than Parmbsc1.

We do not report results for CHARMM36 since for this force field we obtained variations in the free energy profiles of several *k*_*B*_ *T* across runs (see S1 Fig). In several runs with convergence problems we saw that backbone was elongated and deformed, often in combination with stacking of the target base pair (see section Base pair stacked state). Sometimes also neighboring base pairs were affected. Presumably, the difference between CHARMM27 and CHARMM36 is due to the increased backbone flexibility of the latter. Interestingly, a seemingly small change in force field parameters can have great impact on the sampling, especially when it is biased towards high free energy regions as in our case. Similar backbone deformations and stacking structures have also been observed in a free, 90 *μ*s long CHARMM36 simulation [17]. Sampling such structures properly would likely require a more complex reaction coordinate and an amount of sampling beyond the scope of this study.

### Opening mechanisms

**Fig 6:**
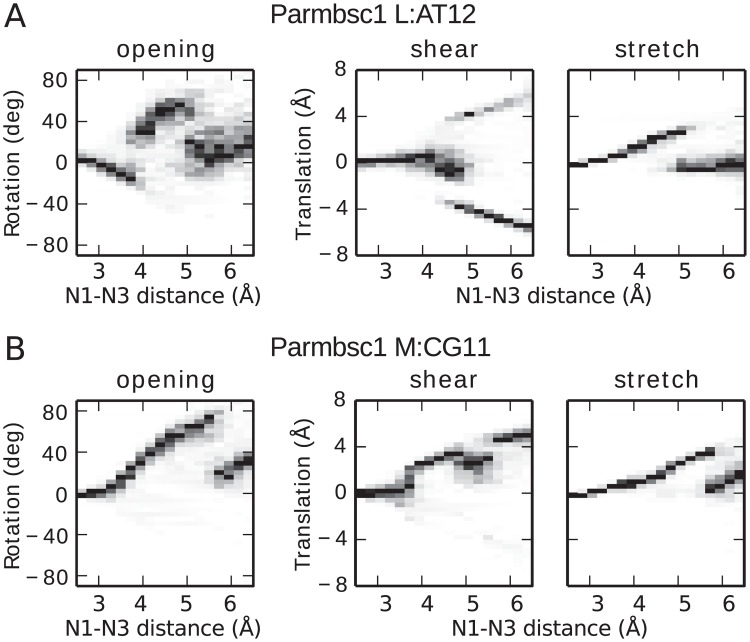
Local base-pair parameter histograms along *d*_N1N3_. The data is from all Parmbsc1 simulations of base pairs L:AT12 (A) and M:CG11 (B), biased along *d*_N1N3_. The histograms have been normalized by dividing by the maximum count along each distance slice. Note that no timeline is implied from left to right.

To further structurally characterize the opening, we analyzed the data in terms of local base-pair parameters, i.e. three translations: shear, stretch, stagger, and three rotations: buckle, propeller and opening, using the 3DNA software [45]. Note that the local base-pair “opening” parameter is different from the dihedral angle *θ* introduced earlier (Fig 1A). They are both angles but for instance *θ* is defined for each base while the opening parameter is defined for one base pair. We plot histograms of opening, shear and stretch as a function of *d*_N1N3_ in Fig 6, again using Parmbsc1 L:AT12 and M:CG11 as illustrative examples. See S2 Fig for histograms of remaining parameters and target base pairs, for both force fields. These types of histograms help us understand what the sampled configurations are, but one should remember they have no dynamic information. For instance a “sudden jump” in the parameter value at a given *d*_N1N3_ value simply corresponds to a free energy barrier along the parameter for that distance. Thus, it does not imply any kind of discontinuity in the trajectories generating the histograms. For instance, major or minor groove opening corresponds to separate pathways which in our simulations would be sampled in a continuous manner by traversing the *d*_N1N3_ sampling interval more than once.

Generally both force fields have similar parameter profiles for small opening distances where the WC hydrogen bonds are still not fully broken while at larger distances the results tend to diverge. For AT opening (Fig 6A) and distances below ∼4 Å, both force fields display increasingly negative opening values, which corresponds to both bases opening toward the minor groove (structure not shown). This allows the major groove WC hydrogen bond to remain intact. This trend is further accompanied by increasingly positive stretch and negative stagger and propeller (see S2 Fig). In the barrier region around *d*_N1N3_ ∼ 4 Å the AT opening mode switches as the major groove hydrogen bond breaks, enabling positive opening in this direction. In a transition region 0.4–0.5 Å, typically both bases contribute to the positive opening value by cooperatively swinging toward the major groove, see Fig 4B. Decreased distances between the hydrogen of A:C2 and T:O2 further help stabilize such configurations. For larger *d*_N1N3_ only one of the bases flips out further while its partner contributes negatively to the opening parameter by shifting to the minor groove instead of following into the major groove, which leads to a drop in the opening parameter value. In the same region shearing increases, negatively or positively depending on which base swings in which direction, enabling the non-WC base pair interactions shown in Fig 4C and 4D.

For GC pairs, opening occurs toward the major groove, i.e. with positive values of the opening parameter (Fig 6B). The major groove hydrogen bond breaks already for *d*_N1N3_ <3.5 Å enabling the rotation of the bases relative to each other. Increasing shear enables the hydrogen acceptor C:O2 to be “shared” between the two donors G:N1 and G:N2 for *d*_N1N3_∼ 4–5 Å (Fig 4F). Likely, these interactions help explain the plateau seen in the corresponding free energy profiles of parmbsc1 (Fig 3). The minor groove hydrogen bond between G:N2 and C:O2 remains intact up until *d*_N1N3_ ∼5.5 Å, acting as a “hinge” for the rotation (Fig 4G). After the last WC hydrogen bond breaks, the base pairs stop directly interacting (Fig 4H). Opening and stretch values drop and larger opening distances are obtained by further increase in shearing and opening values.

The local base-pair parameters are only sensitive to the orientation of one base relative to its partner but do not give information about their orientations relative to the helix backbone. In order to determine which base tends to flip more and into which groove we instead use the dihedral angle *θ* (see Fig 1A). To get an idea of the opening motion in terms of *θ* it is instructive to analyze the histograms *θ*(*d*_N1N3_). We refer the reader to S3 Fig for these histograms. These figures confirm our previous observations that in a transition region typically both bases open up toward the major groove (as in Fig 4B and 4G), while for larger base separation, one base tends to “fall back” into the helix (*θ* ≈ 0°).

To gain more quantitative knowledge about the modes of opening in terms of *θ* however, we decomposed the opening probability into disjoint opening modes. An “opening mode” is here defined by the opening direction for each base, i.e. is *θ* in the major, minor or neither groove? Here, we define the minor and major groove as the intervals *θ* < −Δ*θ* and *θ* > +Δ*θ*, respectively, where Δ*θ* = 30°. Thus, we can map each open configuration into either of 9 opening modes (3 opening directions to the power of 2 bases in a pair). It is clear that the “preferred” mode is sensitive to the choice of Δ*θ*. If it is set to zero, all positive angles will be labeled as major and all negative angles as minor and there will be no distinction between the base being flipped out or not. If it is set too large, there will also be no separation since no configuration will be considered flipped.

The free energy of each mode was calculated by the same reweighting technique as the opening free energy. In Table 1, we report the most probable opening modes for each target base pair, listed in order of preference. We only list modes that are within 1 *k*_*B*_ *T* of the minimum free energy mode. The table shows that when at least one base has |*θ*| > 30°, the clearly dominant modes are those where only one base opens toward the major groove. The preference for the major groove is consistent with previous simulation studies of C flipping [25] while it does not support that both grooves are accessible for pyrimidines as has been proposed in the past [23, 47]. The neither-groove mode ‘–’ is also frequently listed in the table. For parmbsc1 it is even in most cases ranked as the most important mode. This means that even opening at a small angle is sufficient for significantly exposing the imino proton to solvent. In addition, there are clear sequence effects, especially for parmbsc1.

**Table 1:** Preferred opening modes.

Triplet	Base pair	CHARMM27	parmbsc1
TTT	L:TA10	A	A
ATT	L:TA9	A, T	–, T, A
	L:TA13	A, T, –	–
TTA	L:TA11	A, –	–, T
ATA	L:AT8	A, –	A, –, T
	L:AT12	A, –	–, T, A
TTG	L:TA15	A, –	–, T
	M:TA15	A, –	–, T, A
TTC	M:TA10	A, –	–, T
ACA	M:GC8	C, –, G, Cm	–, C
TCT	M:CG11	C, –, Cm	C, –

The opening probablity for each target base pair is split into disjoint opening modes. Each mode is characterized by the direction of both bases in terms their dihedral angles *θ*. In this context, a cutoff of ±30° separates the modes and defines what is meant by major or minor groove opening. All opening modes within 1 *k*_*B*_ *T* of the most probable mode are listed in order of preference, separated by commas. If a base is listed it means the base is facing the major groove (*θ* > 30°) in that mode. Or, if the suffix’m’ is added after the base name it means it is facing the minor groove (*θ* < −30°). No listed base, ‘–’, means that neither base is in either groove.

In case of the GC base pairs, the force fields agree on C being the opening base in agreement with evidence that purine bases stack better than pyrimidine base. Obviously, we cannot exclude the possibility that G is preferred for GC in other sequence contexts. For AT base pairs, the picture is more complex presumably since T is the most hydrophobic base. Both force fields agree that for T flanked by T, i.e. for L:TA10, A is the preferred base to open. More generally however, for AT targets the force fields give different results which is surprising considering the consistent free energy trend we observed for AT base pairs flanked by A or T (Fig 5). In particular, opening base T is more often preferred for parmbsc1 than for CHARMM27. This may be due to underestimation of the hydrophobicity of T from the force field [48]. Possibly it is for the same reason that parmbsc1 displays more sequence sensitivity for these base pairs.

We expect interactions with water molecules to play an important role in the opening mechanism. For instance, when studying the distributions of waters around the open base pair we typically see well-defined clusters of waters molecules hydrogen bonding to atoms of both bases in the open pair (see Fig 4 for examples). Such water bridges have previously been observed in both free simulations [47] and umbrella sampling simulations [49] where they were associated with long-lived opening events and increased water residence times, respectively. From ∼ 200 free simulations of parmbsc1 L:TA10 and M:CG11 starting from the sampled open configurations we estimated the hydrogen bond lifetimes of water with each donor/acceptor atom on both DNA bases, see atom labels in Fig 4, by counting the number of different hydrogen bonding water molecules. Each simulation was continued at least until the open base pair closed, here meaning *d*_N1N3_ < 3 Å. Since we expect different solvation patterns for different states, we split each trajectory into one part with times before closing and another with the remaining times and analyzed them separately. In the case of AT, we further divided the simulations in two groups based on the starting configuration having either A or T more swung out into the major groove, c.f. Fig 4C and 4D.

For the closed state, we find that the water hydrogen bond lifetime at donor/acceptor sites facing the major groove are on the order of 10-100 ps while minor groove facing sites have longer timescales, on the order of ∼ 100 ps. For the central sites A:N1 and T:N3, which become hydrated in the open AT pair, we see a dramatic effect on the water hydrogen bonding lifetimes depending on which groove they face. When A:N1 faces major (Fig 4C) the estimated lifetime is 70 ps. When it turns toward minor (Fig 4D) it increases to 1000 ps. Often, only one or a few waters have time to interact before the base pair closes, which happens on average after 6 ns. Analogous results hold for T:N3. We expect these long-lived interactions to be due to minor groove bridging waters as shown in Fig 4C and 4D. Indeed, in these simulations there is a water molecule within 3.5 Å of both the central site facing the minor groove and its partnering site ∼ 80% of the open time.

For the CG central sites, we obtain water hydrogen bond lifetimes of 90 ps for C:N3. For G:N1 we obtain 400 ps, which is larger than what we typically observe toward the major groove or in bulk water but less than the longest times observed in the case of AT. We note that the closing time limits the maximum hydrogen bonding time we can measure from these simulations, but in the case of CG the average closing time of 3 ns is still an order of magnitude larger. A fundamental difference between the AT and CG pair is that for CG the central and minor groove sites are either both donors (G) or both acceptors (C) while for AT the bases have alternating acceptor/donor sites. Thus, when C is open toward the major groove and a water bridge has formed between the pair, a small rotation or translation of the water molecule could be enough to instead form another water bridge with the neighboring site. This may increase the mobility of the bridging water molecule. For AT on the other hand, for one water bridge to transform into another would require rearrangements also of the DNA. In addition, ion-DNA interactions (see analysis below) could be competing with bridging waters. A more detailed analysis of the water hydrogen-bonding network around the open base pair would be necessary to fully understand these effects.

Binding of Na^+^ to the major groove has been associated with base pair opening in *μ*s long free simulations [17, 49]. In general, the ion composition could change the free energy landscape, as NMR experiments indicate [50]. To investigate such effects, we have analyzed ion, here Na^+^, interactions with the DNA bases for the same set of simulations as for the water analysis. Contact frequencies were obtained using a distance cutoff of 3.5 Å. Ion residence times were calculated as the time interval between the first and last time of contact.

In certain cases and at specific sites, marked by a ’+’ in Fig 4, the contact probability is substantially larger in the open state than after closing. For CG, all three acceptor sites involved in WC bonds (G:O6, C:N3, C:O2) are in contact with Na^+^ 40–50% of the time in the open state. After closing, the major groove acceptor atoms on G (G:O6 and G:N7) were in contact with Na^+^ roughly 15% of the time while the remaing sites have a fraction of less than 3%. The average residence times were generally on the order of ∼ 1000 ps. These observations strongly suggests that ions could play a role in the base pair opening mechanism: destabilizing WC bonds enabled by a fairly common and long-lived interaction with major groove acceptor sites on the base, and preventing closing by occupying WC bonding sites. Also for the open AT base pair in the case where A opens toward major (Fig 4C), the acceptor T:O2 is in contact with Na^+^ 48% of the time. By visual inspection we have verified that this ion is also often in contact with the oxygen of a water bridging from T:N3 to A:N3 (also shown in the figure). On the other hand, when T is open toward the major groove (Fig 4D) we do not observe contact frequencies of the same magnitude.

### Comparison to NMR experiments

Here we address the question: can our simulations reproduce the experimental trend of opening free energies? As was discussed in detail in section Estimating the opening free energy, our calculated free energies are not expected to exactly match NMR values. We would however expect to see the same trends, at least in clear-cut cases. Our statistical accuracy is typically ∼0.2 *k*_*B*_ *T* which is sufficient to distinguish the experimental free energy trend.

Turning again to Fig 5, we see that certain experimental trends are roughly reproduced by simulations. In particular, both force fields assign a relatively high free energy to the GC targets and to L:TA10. However, there are clear discrepancies. For instance, the free energy differences between GC and AT targets are several *k*_*B*_ *T* smaller in the simulations than expected from experiments. Also, in general the experimental free energies are higher than the calculated ones.

In the cases of L:TA15 and M:TA15, which have the lowest free energies according to NMR, the calculated free energies are relatively high for both force fields. One may speculate that there are long timescales especially affecting the ends of base pairs which are beyond our simulation time-scale and that the added end restraints makes it more difficult to open these pairs. To investigate such effects further we calculated the free energy for parmbsc1 M:TA15 with free ends. Intuitively, we would expect this to lead to a lower free energy since the purpose of the end restraints are to stabilize the system. However, freeing the ends resulted in ∼0.3 *k*_*B*_ *T* higher value and an error of the same magnitude. Thus, if there are important long timescale end effects we would need even longer simulation times to probe them.

In the NMR experiments the free energy is derived from average imino proton exchange rates under the assumption of a two-state model, thus assuming a single open state. With MD simulations we can directly measure closing rates. We have done this by choosing a representative set of 165–200 open conformations for two cases, removing the *d*_N1N3_ restraint and measuring the time it takes for the central WC hydrogen bond to reform. In a two-state system the distribution of the open state lifetimes is exponential. In contrast, by applying multi-exponential fitting to our data we obtain two lifetimes of 4 and 20 ns for L:TA10 and of 2 and 7 ns for M:CG11. If there are indeed two lifetimes that differ by factor of 5, there is a possibility that NMR measurements are effectively not detecting all states. In NMR experiments the proton exchange rate is measured as a function of increasing exchange catalyst concentrations until the rate levels off. If there are multiple states with different exchange kinetics, there will be several plateaus in the exchange rate profile. When the concentration is not increased beyond the first plateau, the total closing rate will be underestimated and the free energy overestimated.

A final remark concerns differences in the exchange kinetics of an accessible imino proton in the helix versus a free nucleotide. In the experimental study [6] we are comparing with here, these are considered to be same. Other works assume a difference of a factor 1.5 [51] which accounts for the fact that a base in the helix does not diffuse, unlike a free nucleotide. The simulations reveal an additional difference which is not considered in the experimental studies of proton exchange. In the free nucleotide the imino proton has a hydrogen bond lifetime with water of 10 ps. For AT pairs in the helix with the proton accessible we measured water hydrogen bond lifetime of 100–150 ps, which is an order of magnitude larger. Similar times have been observed for a bridging water in simulations [47] and water in the major groove in NMR experiments [52]. Under the assumption of a two-state model, the result of lower exchange rates would be a lower closing rate as well as a lower opening free energy to account for the same NMR data, but it is unclear how much lower they would be.

### Base pair stacked state

In our trial CHARMM27 AWH-biased simulations we repeatedly observed configurations having the target base pair partners shifted along the helical axis relative to each other and stacked on top of each other. Often the whole *d*_N1N3_ interval was traversed several times before discovering the base pair stacked state after which the system appeared to get “stuck”, sometimes for the remaining simulation time. Our conclusion is that there are free energy barriers associated with transitioning to/from the base pair stacked state which are not being flattened efficiently by the applied bias along the reaction coordinate.

In order to characterize the stacked state further we performed additional AWH simulations on CHARMM27 L:TA10, this time biased along two dimensions: *d*_N1N3_ and the distance between the six-member ring of each base in the pair. Small ring distances can only be obtained by tilting the plane of one ring relative to the other or stacking of the rings. Thus, by biasing toward small ring distances we can enhance sampling of base pair stacked configurations. The two-dimensional sampling region was implicitly defined by setting a maximum free energy difference as has been described previously [18].

**Fig 7:**
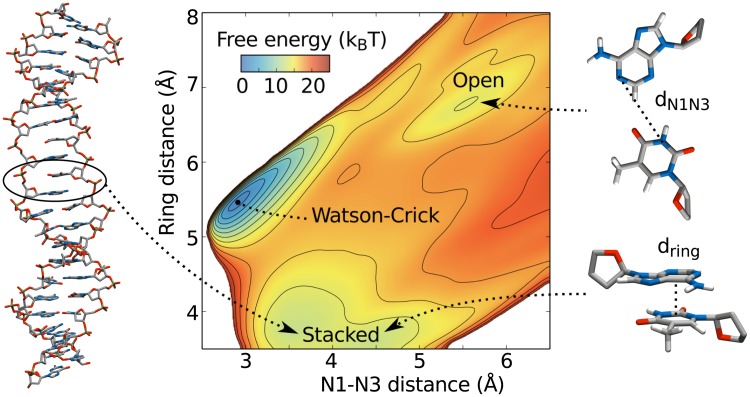
Free energy landscape of *d*_N1N3_ and the six-member ring distance. The definitions of both coordinates are indicated in the figure.

The resulting free energy is shown in Fig 7, an average of 8 independent 290 ns long simulations. The landscape clearly separates three regions of interest: the WC global minimum at (*d*_N1N3_, *d*_ring_)∼(2.9,5.5) Å, a local minimum at ∼(5.5, 6.7) Å, and a relatively populated region *d*_ring_ ≤ 4.6 Å corresponding to the base pair stacked state. The stacked state significantly contributes to the opening free energy. Excluding such configurations, defined as *d*_ring_ < 4.6 Å, from the free energy calculations raises the opening free energy by roughly 1 *k*_*B*_ *T* in this case.

As a further reference, we ran a set of umbrella sampling simulations for the same base pair using the previously well-studied CPDb dihedral angle [24] as a reaction coordinate (see S1 Text for simulation details). Interestingly, also with this reaction coordinate and sampling method we observed base pair stacking in three of the simulations. This proves that the stacked state is not specific to our choice of reaction coordinate or sampling method. It also demonstrates that when using umbrella sampling, looking at the obtained free energy profile and its error bars (see Fig in S1 Text) is generally not sufficient for detecting sampling issues of this sort.

To our surprise, we have not been able to find previous reports on similar observations of base pair stacking for CHARMM27. One possibility is that such configurations have been sampled but not detected. Alternatively, previous authors may have been “lucky” enough to not sample the stacked state, for instance because of less extensive sampling than in the present study.

Without further analysis we cannot assess whether the base pair stacked state is real or the result of the force field, CHARMM in particular, being poorly parameterized in this high free energy region of phase space. In this work we have for sampling reasons effectively assumed the latter by adding to all our simulations a bias potential designed to prevent the system from sampling below a certain value of *d*_ring_. The potential was added for both CHARMM and parmbsc1 runs. For parmbsc1 simulations however *d*_ring_ rarely goes below the cutoff and so the added bias is effectively not applied.

### Conclusions

We have investigated the sequence dependency of DNA base pair opening using atomistic MD simulations. Both GC and AT opening have been targeted in two different sequences and using two force fields: CHARMM27 and parmbsc1. We have focused sampling on small base pair openings, including only the most probable regions where the imino proton is exposed to solvent. In this region, the distance *d*_N1N3_ has been demonstrated to be an effective biasing reaction coordinate which makes minimal assumptions about the opening pathway.

We have obtained free energy profiles along *d*_N1N3_ with a statistical accuracy of 0.2–0.3 *k*_*B*_ *T* using a robust adaptive biasing method, AWH. For opening of AT base pairs we have characterized two important interactions between the opening bases contributing to a local minima along the free energy profile. In addition, in the case of one GC and one AT base pair, we have shown that the open base pair provides specific sites in the major groove where water bridges between the bases can form and where the water-DNA hydrogen bonding time is on the 1000 ps and 100 ps timescale for AT and GC, respectively. For the same base pairs we have shown that certain acceptor atoms are very likely to be in contact with Na^+^ in the open configurations.

We have also calculated opening free energies, where “open” here means that the imino proton is hydrogen bonded with water. By determining the free energy contribution of different opening modes, we have shown that opening typically occurs by flipping one base >30° into the major groove while the other base remains within the helix (<30°). The opening pathway is however not as simple as one base flipping independently of its WC partner. For example, for AT opening it is likely to find both bases slightly perturbed towards the major groove in the free energy barrier region. In addition, configurations where both bases open less than 30° contribute significantly to the opening free energy.

Furthermore, we have shown that both the free energy and the preferred opening mode are sequence dependent. In particular, the nearest neighbor sequence is a dominant factor. However, we have also observed differences for base pairs with the same nearest neighbors indicating the presence of more distant sequence effects. The two force fields reproduce the same free energy trend for most of the target base pairs. However, for opening of AT pairs parmbsc1 tends to favor flipping of T more than CHARMM27.

Compared to free energies obtained from NMR experiments, the values calculated here are generally lower. The interpretation of NMR proton exchange experiments has, necessarily, relied on simple models such as two-state kinetics. The simulations show that this is probably an overly simplified picture of reality. Depending on the sequence there can be multiple open states and, as has been shown previously, water molecules can bridge the opening base pairs which can affect free energies and proton exchange.

The extensive amount of sampling together with the use of a sampling method allowing for multiple pathways has revealed the existence of a base pair stacked state in which the WC partners stack their six-membered rings on top of each other. We observe base pair stacking for both force fields, but to a very different extent; for CHARMM27 it is relatively low in free energy and thus complicates sampling, whereas for parmbsc1 it only occurs rarely and transiently. Thus, we cannot exclude the possibility that this is a force field issue. Alternatively, if it is physically relevant, our simulations show that base pair stacking could contribute significantly to NMR opening free energies.

The present work has demonstrated the potential of studying base pair opening with advanced simulation methods. Our results show how sequence affects the free energy, modes and mechanism of base pair opening, only the first of which is accessible by NMR experiments. This information is essential for understanding how molecules interact with DNA.

## Supporting information

S1 Fig CHARMM36 L:TA10 free energy profiles. Non-converged free energy profiles along *d*_N1N3_ for CHARMM36 L:TA10.
(PDF)

S2 Fig Local base-pair parameter histograms. Histograms of the local base-pair parameters as a function of *d*_N1N3_ for all force fields, base pairs and parameters.
(PDF)

S3 Fig Dihedral angle histograms. Histograms of the opening dihedral angle as a function of *d*_N1N3_ for all force fields, base pairs and both bases.
(PDF)

S1 File Template input parameter file for GROMACS. Template file with GROMACS settings for CHARMM or Parmsbsc1 simulations, with or without AWH biasing.
(PDF)

S1 Appendix Details on free energy calculations. Derivation and details of the equations used for calculating free energy averages and error estimates.
(PDF)

S1 Text Umbrella sampling setup and results. Simulation details and results for the umbrella sampling simulations.
(PDF)
