# Supplementary material for: Sequence dependency of canonical base pair opening in the DNA double helix
Source: PLoS Comput Biol. 2017 Apr 3;13(4):e1005463. doi: 10.1371/journal.pcbi.1005463 (PMC5393899; doi:10.1371/journal.pcbi.1005463)
Supplement: S1 Fig — Non-converged free energy profiles along dN1N3 for CHARMM36 L:TA10. (PDF) [file pcbi.1005463.s001.pdf]

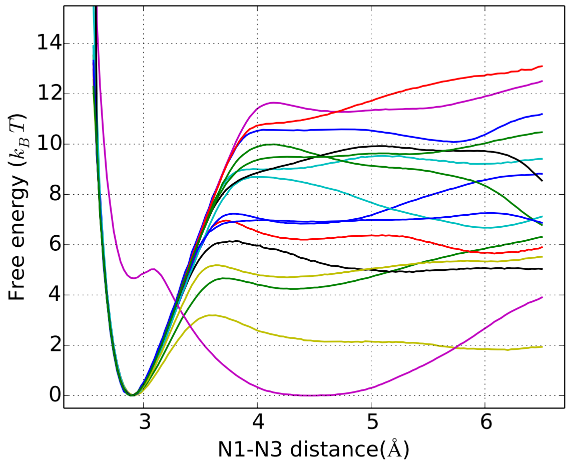

Fig S1.A. Free energy profiles for CHARMM36 L:TA10. Each of the 16 profiles (marked with different colors) was calculated from a 100 ns long, independent AWH biased simulation. Clearly, the results are not converged. Other target base pairs were not simulated for this force field.
