# Supplementary material for: Sequence dependency of canonical base pair opening in the DNA double helix
Source: PLoS Comput Biol. 2017 Apr 3;13(4):e1005463. doi: 10.1371/journal.pcbi.1005463 (PMC5393899; doi:10.1371/journal.pcbi.1005463)
Supplement: S2 Fig — Histograms of the local base-pair parameters as a function of dN1N3 for all force fields, base pairs and parameters. (PDF) [file pcbi.1005463.s002.pdf]

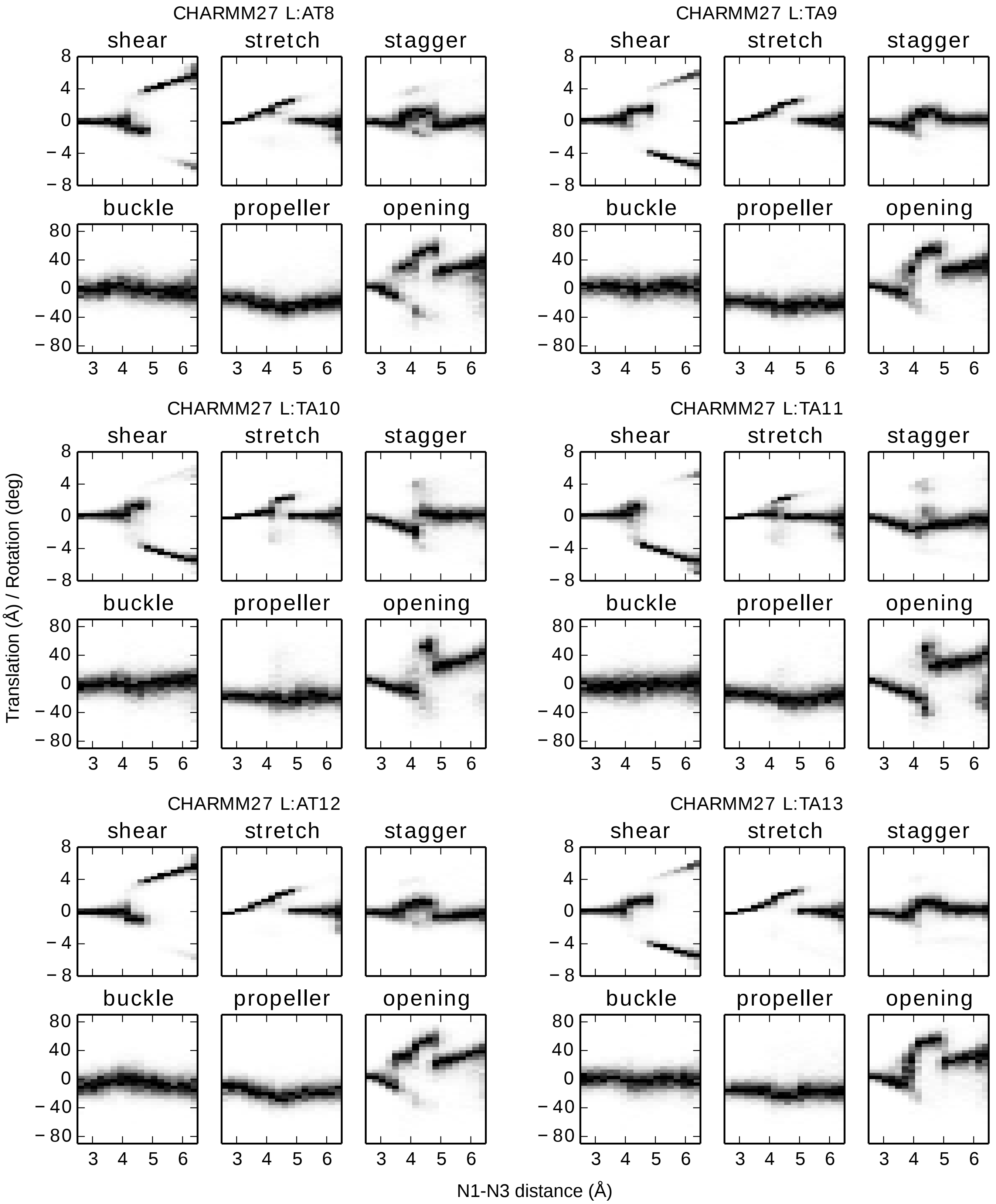

Fig S2.A. CHARMM27 local base-pair parameter distributions as a function of $d_{\mathbf{N1N3}}$.

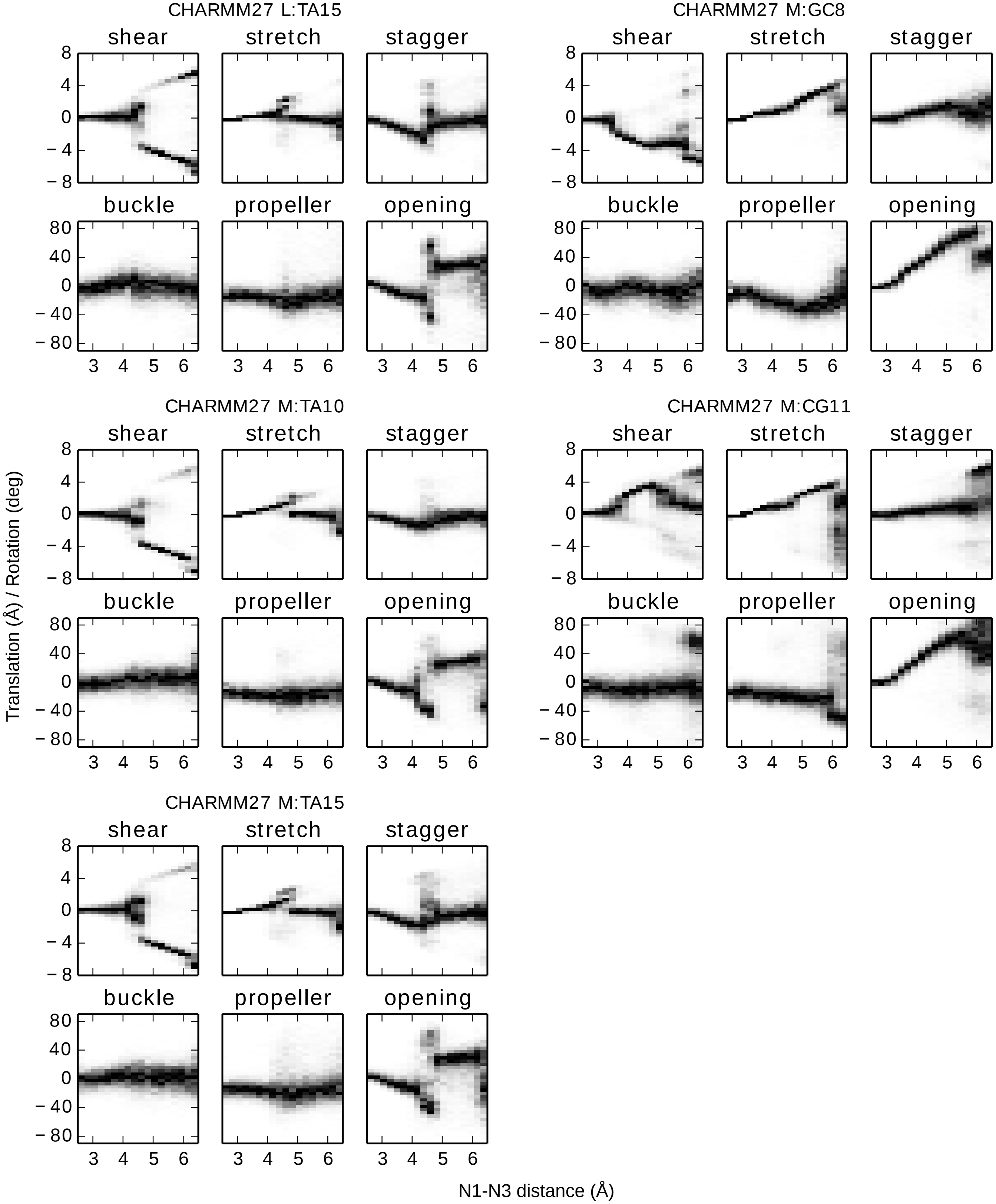

Fig S2.B. CHARMM27 local base-pair parameter distributions as a function of $d_{\mathbf{N1N3}}$.

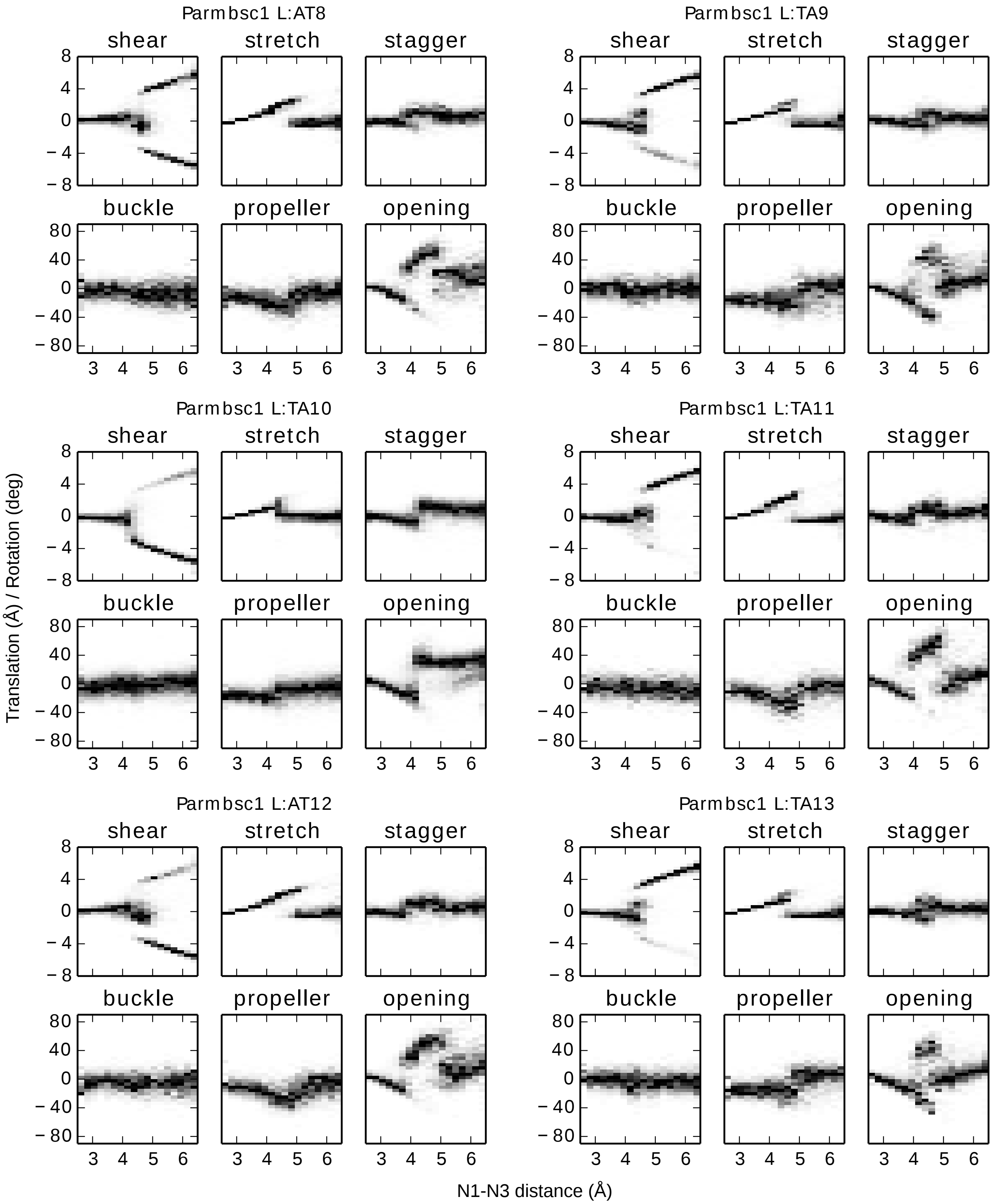

Fig S2.C. Parmbsc1 local base-pair parameter distributions as a function of $d_{\mathbf{N1N3}}$.

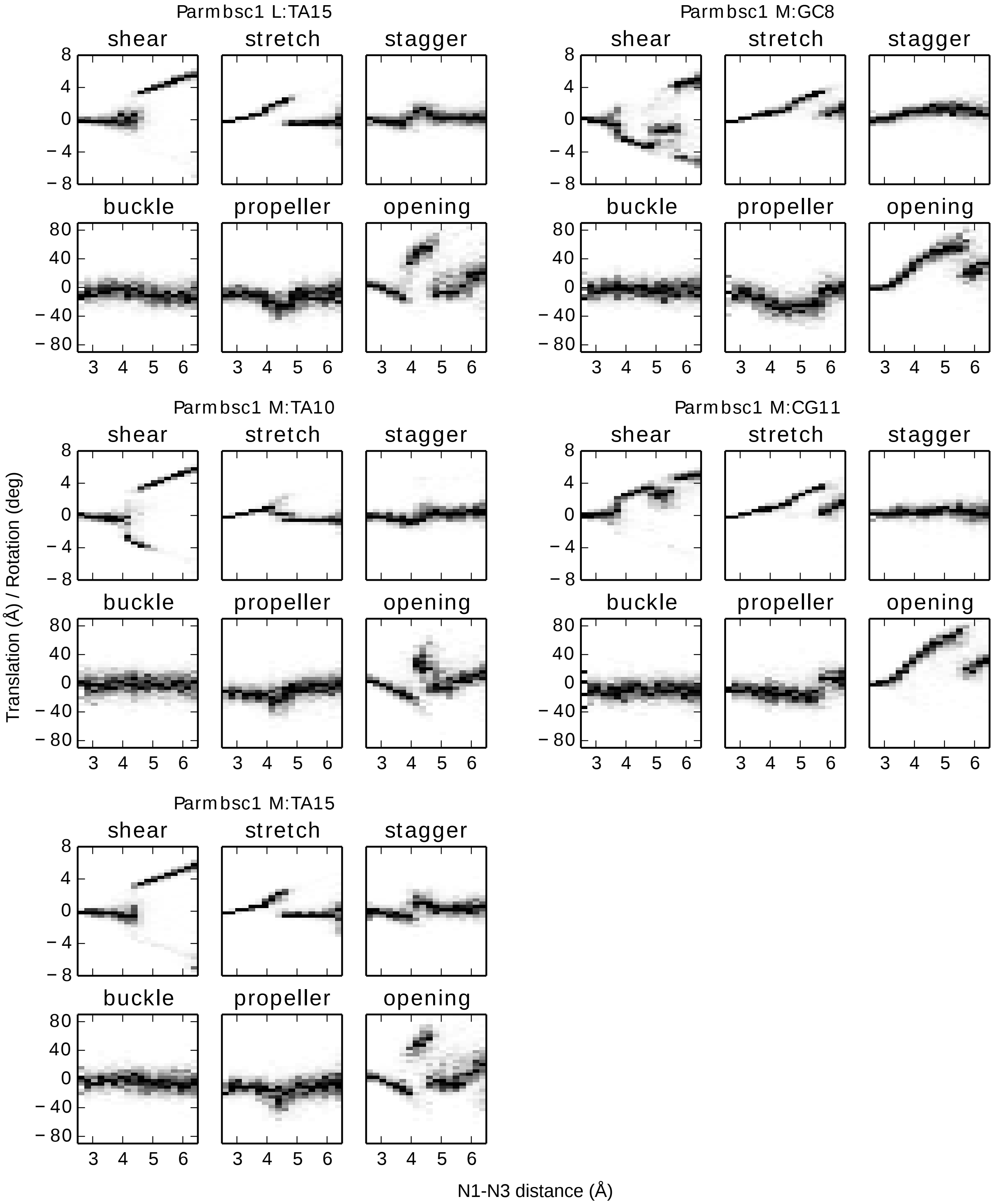

Fig S2.D. Parmbsc1 local base-pair parameter distributions as a function of d_{N1N3}.
