# Supplementary material for: Sequence dependency of canonical base pair opening in the DNA double helix
Source: PLoS Comput Biol. 2017 Apr 3;13(4):e1005463. doi: 10.1371/journal.pcbi.1005463 (PMC5393899; doi:10.1371/journal.pcbi.1005463)
Supplement: S3 Fig — Histograms of the opening dihedral angle as a function of dN1N3 for all force fields, base pairs and both bases. (PDF) [file pcbi.1005463.s003.pdf]

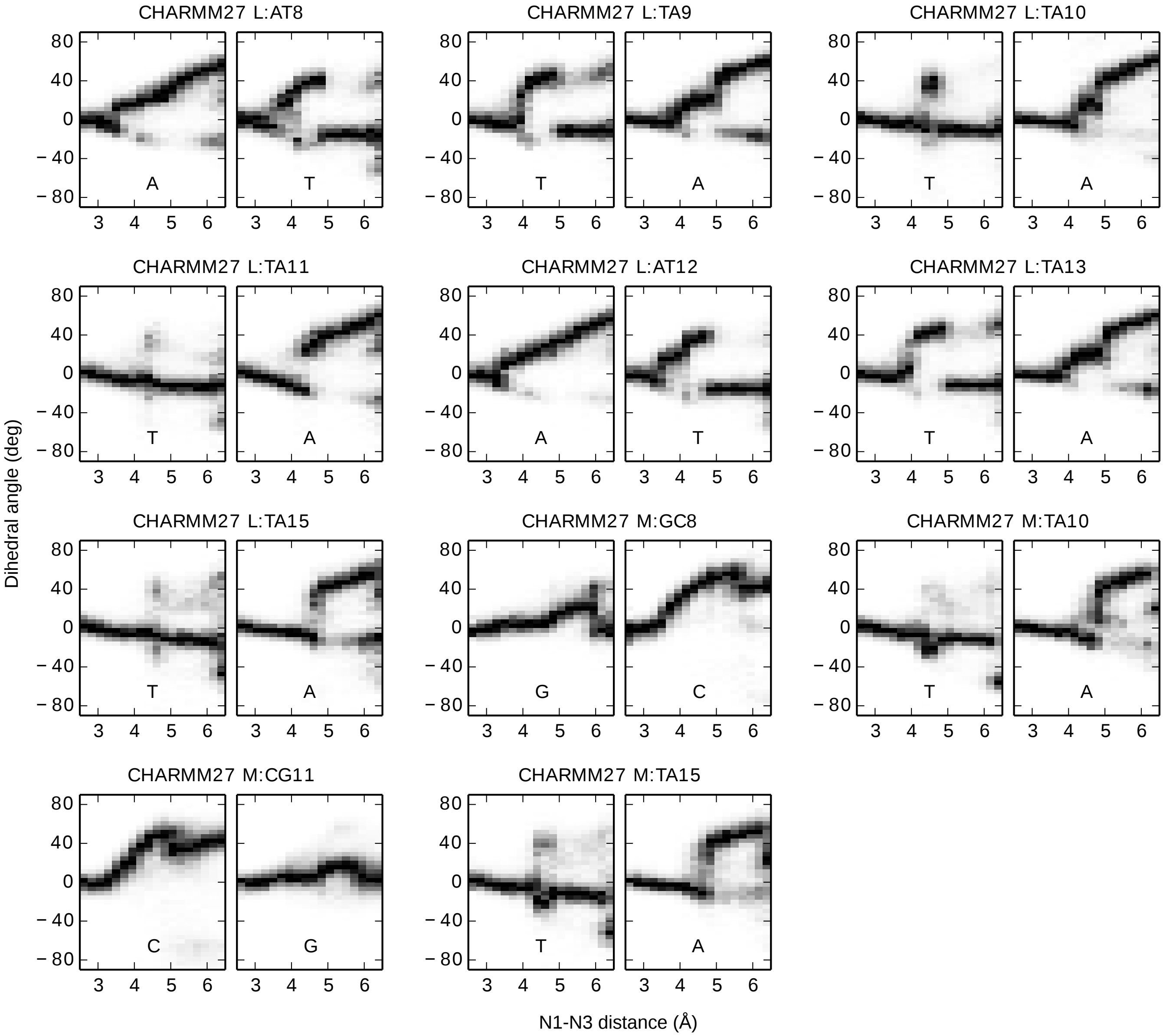

Fig S3.A. CHARMM27 dihedral angle distributions as a function of $d_{\mathbf{N1N3}}$.

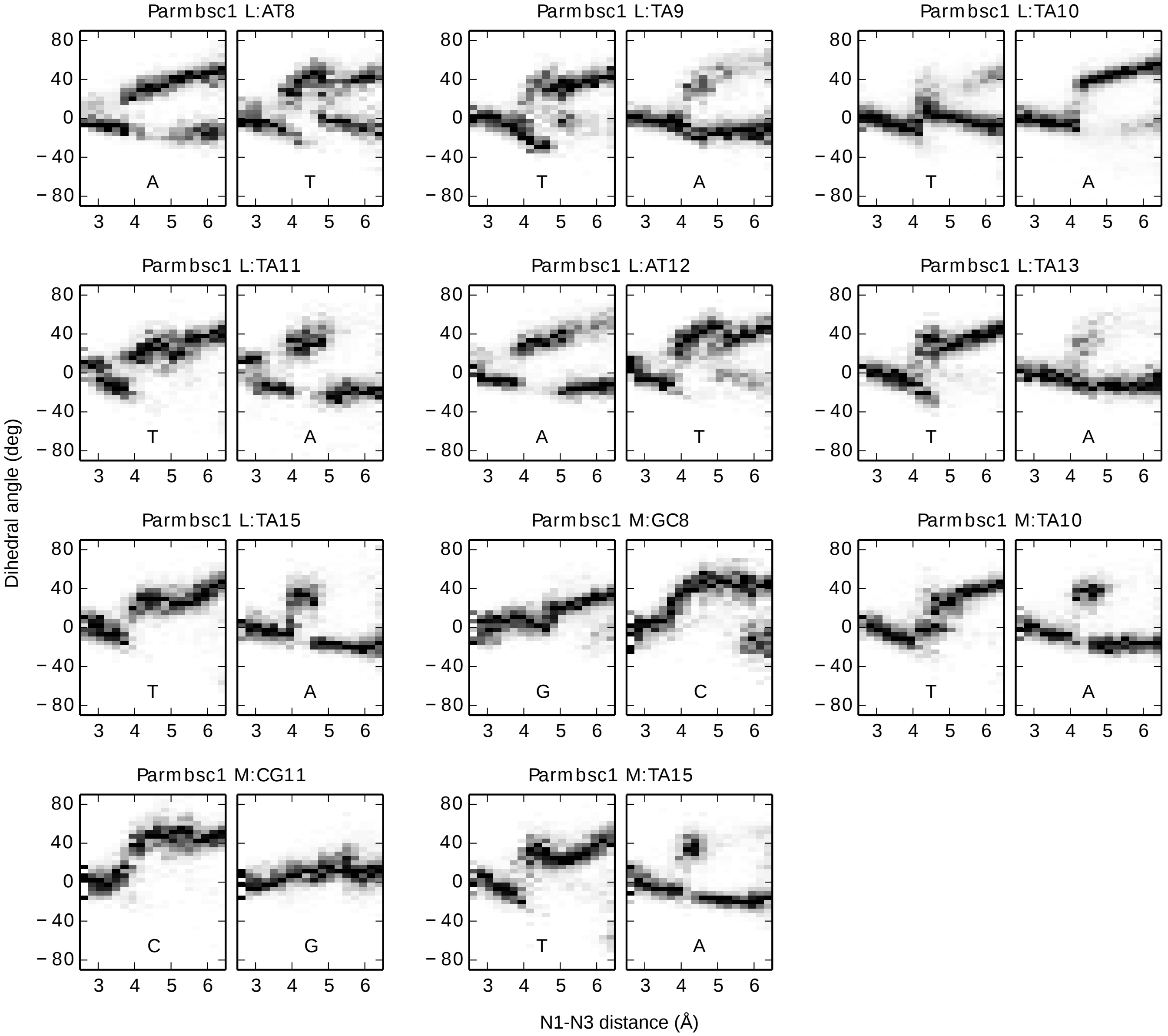

Fig S3.B. Parmbsc1 dihedral angle distributions as a function of $d_{\mathbf{N1N3}}$.
