# Supplementary material for: Sequence dependency of canonical base pair opening in the DNA double helix
Source: PLoS Comput Biol. 2017 Apr 3;13(4):e1005463. doi: 10.1371/journal.pcbi.1005463 (PMC5393899; doi:10.1371/journal.pcbi.1005463)
Supplement: S1 File — Template file with GROMACS settings for CHARMM or Parmsbsc1 simulations, with or without AWH biasing. (PDF) [file pcbi.1005463.s004.pdf]

S5 File. Template Input Parameter File for GROMACS

```
; Use for CHARMM or Parmbsc1 by commenting out or
; uncommenting the relevant lines.
;-------------------------------------------------------
integrator               = md
dt                       = 0.002
nsteps                   = 50000000
nstxout                  = 0
nstvout                  = 0
nstfout                  = 0
nstlog                   = 5000000
nstenergy                = 50000
nstxtcout                = 50000
comm_grps                = system
xtc_grps                 = system
energygrps               = DNA SOL
cut-off-scheme           = Verlet
nstlist                  = 10
ns_type                  = grid
pbc                      = xyz
coulombtype              = PME
coulomb-modifier         = Potential-shift-Verlet
ewald-rtol               = 1e-5
pme-order                = 4
rcoulomb                 = 1.2             ; CHARMM
fourierspacing           = 0.14            ; CHARMM
;rcoulomb                = 1.0             ; Parmbsc1
;fourierspacing          = 0.125           ; Parmbsc1
vdwtype                  = Cut-off
vdw-modifier             = Force-switch    ; CHARMM
rvdw-switch              = 0.8             ; CHARMM
rvdw                     = 1.2             ; CHARMM
DispCorr                 = No              ; CHARMM
;vdw-modifier            = Potential-shift ; Parmbsc1
;rvdw-switch             = 0               ; Parmbsc1
;rvdw                    = 1.0             ; Parmbsc1
;DispCorr                = EnerPres        ; Parmbsc1
constraints              = h-bonds
constraint_algorithm     = LINCS
lincs_iter               = 4
Pcoupl                   = Parrinello-Rahman
tau_p                    = 5.0
ref_p                    = 1.0
compressibility          = 4.5e-5
Tcoupl                   = v-rescale
tc-grps                  = system
tau_t                    = 0.5
ref_t                    = 300
; Comment out pull and AWH parameters for running
; unbiased simulations. Otherwise, AWH is applied
```

```
; to target base pair L:TA10 along the N1-N3 distance
; and a flat bottom potential is applied to the ring
; distance.
pull                     = yes
pull-print-ref-value     = yes
pull-nstxout             = 5000
pull-nstfout             = 0
pull-ngroups             = 4
pull-ncoords             = 2
pull-group1-name         = r_11_&_N3
pull-group2-name         = r_28_&_N1
pull-coord1-groups       = 1 2
pull-coord1-type         = umbrella
pull-coord1-start        = yes
pull-coord1-geometry     = distance
pull-coord1-k            = 32000
pull-group3-name         = r_11_&_C2_C4_C5_C6_N1_N3
pull-group4-name         = r_28_&_C2_C4_C5_C6_N1_N3
pull-coord2-groups       = 3 4
pull-coord2-type         = flat-bottom-high
pull-coord2-start        = no
pull-coord2-init         = 0.48
pull-coord2-geometry     = distance
pull-coord2-k            = 32000
awh                     = yes
awh-nbias               = 1
awh-nstout              = 50000
awh1-target             = constant
awh1-user-target        = no
awh1-ndim               = 1
awh1-error-init         = 5
awh1-dim1-pull-coord    = 1
awh1-dim1-diffusion     = 5e-5
awh1-dim1-min           = 0.25
awh1-dim1-max           = 0.65
```
