# Supplementary material for: Sequence dependency of canonical base pair opening in the DNA double helix
Source: PLoS Comput Biol. 2017 Apr 3;13(4):e1005463. doi: 10.1371/journal.pcbi.1005463 (PMC5393899; doi:10.1371/journal.pcbi.1005463)
Supplement: S1 Appendix — Derivation and details of the equations used for calculating free energy averages and error estimates. (PDF) [file pcbi.1005463.s005.pdf]

S1 Appendix. Details on Free Energy Calculations

AWH is defined in an extended ensemble with probability distribution

$$P(x,\lambda) = \frac{1}{\mathcal{Z}} \mathrm{e}^{g(\lambda) - E(x,\lambda)}, \tag{A.1}$$

where x is the system configuration, λ is the biasing parameter, $g(\lambda)$ is a tunable (time-dependent) bias function, $E(x,\lambda)$ is the potential describing the system and $\mathcal{Z} = \sum_\lambda \int \mathrm{d}x P(x,\lambda)$ is the extended ensemble partition function that normalizes the probability. Note that P is generally a biased probability because of $g(\lambda)$ and so does not directly represent the "real", unbiased system.

For the case of biasing along a reaction coordinate $\xi(x)$ the original system is biased by a harmonic potential $Q(\xi,\lambda)$ restraining ξ to a reference position λ. The resulting extended ensemble potential is given by $E(x,\lambda) = E_0(x) + Q(\xi(x),\lambda)$, where E_0 is the unbiased potential.

Assume we have data biased along a reaction coordinate $\xi(x)$ but are actually interested in the free energy along another observable $u(x)$. From Eq (A.1) we obtain the joint biased probability of u and ξ

$$\begin{aligned} P(u,\xi) &= \sum_\lambda \int \mathrm{d}x\, P(x,\lambda)\delta(u - u(x))\delta(\xi - \xi(x)) \\ &= \sum_\lambda \int \mathrm{d}x\, \frac{1}{\mathcal{Z}} \mathrm{e}^{g(\lambda) - E_0(x) - Q(\xi(x),\lambda)} \delta(u - u(x))\delta(\xi - \xi(x)) \\ &= \frac{1}{\mathcal{Z}} \sum_\lambda \mathrm{e}^{g(\lambda) - Q(\xi,\lambda)} \cdot \int \mathrm{d}x\, \mathrm{e}^{-E_0(x)} \delta(u - u(x))\delta(\xi - \xi(x)) \\ &= \frac{1}{\mathcal{Z}} \mathrm{e}^{b(\xi)} \cdot \mathrm{e}^{-\Phi(u,\xi)}, \end{aligned} \tag{A.2}$$

where $\Phi(u,\xi)$ is the joint free energy of u and ξ in the unbiased system and $b(\xi)$ is an effective bias function along ξ defined by $\mathrm{e}^{b(\xi)} = \sum_\lambda \mathrm{e}^{g(\lambda) - Q(\xi,\lambda)}$.

The unbiased free energy along u is obtained by integrating over ξ, $\Phi(u) = -\ln(\int \mathrm{d}\xi\, \mathrm{e}^{-\Phi(u,\xi)})$. Thus, using (A.2) we obtain

$$\mathrm{e}^{-\Phi(u)} = \mathcal{Z} \int \mathrm{d}\xi\, P(u,\xi)\, \mathrm{e}^{-b(\xi)}. \tag{A.3}$$

In practice, Eq (A.3) is interpreted as a time average and can be applied post-simulation by histogramming along u and summing up samples from all simulations indexed by i,

$$\mathrm{e}^{-\hat{\Phi}(u)} = \sum_{i,t} 1_u(u_t^i)\mathrm{e}^{-b_t^i(\xi_t^i)}\hat{\mathcal{Z}}_t^i, \tag{A.4}$$

where we here use the same notation as for Eq (1) in the main text. Eq (A.4) is identical to Eq (1) after rewriting the partition functions in terms of the free energy along ξ, $\hat{\mathcal{Z}}_t^i = \int \mathrm{d}\xi\, \mathrm{e}^{-\hat{\Phi}(\xi)+b_t^i(\xi)}$.

For the special case $u = \xi$, Eq (A.3) reduces to

$$\mathrm{e}^{-\Phi(\xi)} = \mathcal{Z}P(\xi)\,\mathrm{e}^{-b(\xi)}, \tag{A.5}$$

which is the formula used for extracting the free energy along the biased coordinate ξ during the AWH simulation.

To consistently combine multiple free energy estimates $\hat{\Phi}^i(\xi)$ obtained from independent simulations we estimate the biased probability $P^i(\xi)$ by the histogram $N^i\mathrm{e}^{-\hat{\Phi}^i(\xi)+b^i(\xi)}/Z^i$ where N^i is the number of samples in simulation i, $Z^i = \int \mathrm{d}\xi\, \mathrm{e}^{-\hat{\Phi}^i(\xi)+b^i(\xi)}$ normalizes the histogram, and $b^i(\xi) = b^i_{t_{\mathrm{final}}}(\xi)$. After summing Eq (A.5) over simulations we so obtain an expression for the average free energy $\hat{\Phi}(\xi)$,

$$\mathrm{e}^{-\hat{\Phi}(\xi)} = \sum_i N^i\mathrm{e}^{-\hat{\Phi}^i(\xi)}\frac{\hat{\mathcal{Z}}^i}{Z^i}. \tag{A.6}$$

Eq (A.6) is identical to Eq (2) after rewriting Z^i and $\hat{\mathcal{Z}}^i$ in terms of $\hat{\Phi}^i(\xi)$.

We estimate the standard deviation of our free energy averages, obtained either by Eq (1) or Eq (2), using jackknifing. Jackknife errors are obtained by deleting observations (here, simulations) one by one and calculating new averages based on these reduced sets. In our case, the jackknife standard deviation of $\hat{\Phi}(u)$, i.e. the standard error of the mean at a given value of u, is given by

$$\sigma(u) = \sqrt{\frac{n-1}{n}\sum_{i=1}^{n}\left(\hat{\Phi}_{-i}(u) - \hat{\Phi}(u)\right)^2}, \tag{A.7}$$

where n equals the number of simulations and $\hat{\Phi}_{-i}(u)$ is the free energy estimate

obtained when excluding simulation i. Since the free energy is only determined up to a constant, clearly the alignment of $\hat{\Phi}_{-i}(u)$ relative to $\hat{\Phi}(u)$ needs to be chosen. Here, we subtract the minimum value, i.e. the minimum free energy is used as a reference.
