# Supplementary material for: Sequence dependency of canonical base pair opening in the DNA double helix
Source: PLoS Comput Biol. 2017 Apr 3;13(4):e1005463. doi: 10.1371/journal.pcbi.1005463 (PMC5393899; doi:10.1371/journal.pcbi.1005463)
Supplement: S1 Text — Simulation details and results for the umbrella sampling simulations. (PDF) [file pcbi.1005463.s006.pdf]

S4 Text. Umbrella Sampling Setup and Results

We performed “standard” umbrella sampling simulations for for CHARMM27 L:TA10 along the CPDb dihedral angle. Note that this dihedral angle has a different definition than that used for the opening mode analysis (see main text) and cannot be directly compared. Positive angles correspond to flipping into the major groove, negative angles to the minor groove. The initial configurations for each umbrella position λ, for both A and T base flipping, were generated by pulling at a constant rate of 180°/ns for 1 ns in both the positive and negative directions starting from the equilibrated system (at -2.6°). We then ran 36 restrained simulations each of length 5 ns in the angle interval ±90°, i.e. using an umbrella distance of 5°. The force constant was 64 000 kJ/(mol $\cdot$ rad^2) for the initial pulling and 6400 kJ/(mol $\cdot$ rad^2) for the final umbrella sampling. The last 4 ns were used for calculating the free energy profile with WHAM, shown in FigS4.A. Base pair stacking occurred for T flipping toward the minor groove for three simulations with umbrella windows in the range of -35° to -20°, in two cases after more than 1 ns of equilibration.

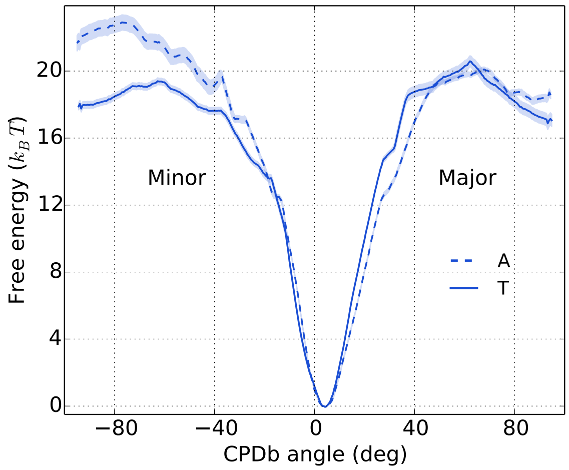

Fig S4.A. Free energy along the CPDb dihedral angle.
